# Sensemaking Processes during the First Months of COVID-19 Pandemic: Using Diaries to Deepen How Italian Youths Experienced Lockdown Measures

**DOI:** 10.3390/ijerph182312569

**Published:** 2021-11-29

**Authors:** Fortuna Procentese, Flora Gatti, Emiliano Ceglie

**Affiliations:** Department of Humanities, University of Naples Federico II, 80133 Naples, Italy; flora.gatti@unina.it (F.G.); emiliano.ceglie94@gmail.com (E.C.)

**Keywords:** COVID-19, pandemic, social relationships, sensemaking, lockdown, coping strategies, arousal, grounded theory, Italian youths, university

## Abstract

The COVID-19 pandemic has brought about disruptive changes in individuals’ lives, breaking the established systems of meaning worldwide. Indeed, in the first months of the pandemic, with individuals being forced to stay at home for a prolonged time to contain the spread of the virus, the need to build new meanings to understand and face this crisis emerged. Building on this, the present study contributes to the understanding of how sensemaking processes were shaped in the face of COVID-19 collective trauma during the very first months of the pandemic. Hence, 36 Italian young adults aged between 21 and 25 submitted daily diary entries for two weeks (T1 was the third week of Italian National lockdown; T2 was the penultimate week before the ease of such stay-at-home orders), resulting in 504 texts. The stimulus was always “Could you describe your daily experience and feelings?”. The Grounded Theory was used. Thus, 15 categories emerged, grouped into three macro-categories. The core category was sensemaking as adaptation. Indeed, the sensemaking process seemed to be a strategy to adapt to the new circumstances related to the lockdown, facing the emotional, cognitive, and activation reactions such conditions by relying on coping strategies and the redefinition of primary as well as broader social relationships.

## 1. Introduction

In the first months of 2020, lots of governments around the world enforced lockdown measures in their countries as an attempt to control the spread of coronavirus disease 2019 (COVID-19) pandemic [1]. Such measures were aimed at reducing the threats to individuals’ health and to the functioning of local sanitary systems [2]. In Italy, which is the context where the present study was carried out, a nationwide lockdown was issued and lasted about two months, between March and May 2020.

Building on the nature of the pandemic and of the measures needed to slow down the contagion trend in its very first phase, COVID-19 has been more than a sanitary and health emergency in individuals’ experience. Indeed, it may be considered a total social fact [3], that is, a phenomenon impacting all the aspects of individuals’ social and personal lives. Consistently, it has been defined as a syndemic [4] and a cultural trauma [5]. Indeed, these measures, as far as needed, required citizens to face disruptive, unprecedented, and unexpected changes in their daily lives and habits [2,6,7,8,9,10,11]. Further, they suddenly changed their opportunities to experience their communities, meant both as physical spaces (e.g., streets and common areas were empty and unavailable due to stay-at-home orders) and as social entities (e.g., a growing mistrust towards others as potential COVID-19 carriers emerged) [12]. Overall, the COVID-19 pandemic and the related lockdown brought about the need to create new individual and collective meanings as well as adaptive responses to these changes [13].

Thus, understanding how the COVID-19 pandemic has shaped personal and collective sensemaking processes represents a timely and worthwhile challenge [14]. Indeed, an extraordinary global event such as a pandemic shakes the foundations of meaning in individuals’ lives [15] and provides individuals with spontaneous liminal experiences [16]. Consistently, it requires the redefinition and reframing of relationships, identities, and individual and shared habits and routines. Indeed, personal and social rhythms had to change [11,12] as an attempt to adapt to and cope with the disruptive changes brought about by the COVID-19 pandemic, as well as to comply with the protective measures enforced by National governments and health authorities. That is, under emergency circumstances, individuals had to face a broad disorientation which was brought about by the loss of the place and time landmarks usually marking their daily routines, as they were no longer allowed to go out of their house if not for groceries. The disruption of their experiences of home, places, relationships, and identities followed [17]: houses were also offices or classes, work, rest, and leisure spaces collapsed into one; friends, colleagues, and relatives out of the households were avatars or pictures on mobile or laptop screens; daily life places were only available to be seen in photographs. Nevertheless, all these elements, as far as experienced in an unusual way, may have also served as “psychological and structural bearings” [17] (p. 401) citizens have relied on in order to identify new boundaries and balances during the months of lockdown. Such bearings may have helped in reconstructing habits and identities in an environment characterized by renewed yet still familiar landmarks [17].

Consistently, this study aims at deepening citizens’ lockdown experience in order to gain a better understanding of sensemaking processes, as well as of the familiar elements that drove adaptation and reorientation processes under these unprecedented circumstances. Specifically, it focuses on young adults’ experiences, since social opportunities and daily activities usually allow them to feel autonomous and independent, which is critical to their evolutionary tasks and identity formation processes [18]. In the face of these broad and persistent stay-at-home orders and of their later ease when risks for citizens’ safety still existed, young adults had to face the need to adapt their sociability, autonomy, and independence, to find new ways to manage their daily activities and relationships [12].

## 2. The Study

Building on this already established knowledge about disorientation and reorientation processes due to small- or large-scale emergencies [17], the present study aims at achieving a deeper understanding of the strategies and processes which served as drivers for reorientation and sensemaking processes as to daily life experiences and feelings under these unprecedented lockdown circumstances. Thus, the first research question leading this study can be expressed as follows:

*RQ1*: What are the relevant strategies regarding reorientation processes during the nationwide COVID-19-related lockdown?

Indeed, it is necessary to mention that the nationwide lockdown was issued in the very first phase of the COVID-19 outbreak, between March and May 2020, which can be characterized as a time in which the virus was not yet known. Consistently, the opportunities to get back to pre-COVID-19 life were felt as still feasible.

Specifically, the strategies compounding reorientation processes under lockdown measures will be understood with reference to two different times: (a) during the third week of lockdown and (b) during the penultimate week before the ease of lockdown measures (National Institutions had already announced this decision to citizens, meaning they were already aware about this being about to happen). This choice was aimed at understanding on which strategies young adults relied in two critical moments which caused disorientation and required reorientation processes: that is, (a) when they were adapting to strict stay-at-home orders and to the sudden loss of all their daily activities and face-to-face relational opportunities, and (b) when they knew that such orders were to be eased in a few weeks, the resumption of daily activities and social opportunities was supposed to gradually happen, yet protective behaviors and high levels of attention were still required of citizens since the pandemic was not over yet.

This study endeavors to frame COVID-19 pandemic and the related emergency according to DeWolfe’s linear model of disaster responses [19] despite COVID-19 representing a quite different kind of emergency. This model is composed of seven phases, namely a warning or threat phase, impact phase, rescue or heroic phase, remedy or honeymoon phase, inventory phase, disillusionment phase, and reconstruction or recovery phase, that follow each other gradually. Specifically, the first time taken into account in this study may be defined as the impact phase (that is, when the emergency broke out, after a time of fear and uncertainty), which had been anticipated by about two months of warnings about the spread of the disease [20]. According to DeWolfe, in this phase, typical reactions are confusion and disbelief, along with a focus on oneself and one’s loved ones’ survival and physical well-being [19]. As regards the second time of this study, several hypotheses about DeWolfe’s phases can be proposed. First, due to the announcement about the ease of lockdown measures to the Italian population, the remedy or honeymoon phases could be expected, since these phases are characterized by optimistic experiences, bonding—even technology-mediated—, and community mutual support [19]. Nevertheless, it is also necessary to mention that lockdown measures persisted enough to generate financial pressure, confinement stress, and other multiple demands (e.g., the availability of digital devices in families, the emotional load due to physical and sometimes social distancing) which are typical of the inventory phase, during which survivors become physically exhausted and optimism could give way to discouragement and fatigue [19]. Further, due to these persistent changes in their daily lives, citizens may have also focused on the gap between their current conditions and pre-emergency ones when thinking about the resumption to their daily life out-of-home—which is typical of the disillusionment phase [19]. Last, still due to the persistent changes they were forced to experience due to stay-at-home orders, citizens may have become aware about the rebuilding of their daily habits and opportunities and the restart of their lives depending on them, which may have brought them to taking responsibilities in order to create new ones. This is rather typical of the reconstruction/recovery phase [19]. For these reasons and due to the unprecedented nature of this emergency, it is not easy to determine to which phase of DeWolfe’s model [19] the second time of this study might be more similar to. Thus, it seems reasonable to hypothesize that the end of strict stay-at-home orders may have produced a mixed phase during the COVID-19 pandemic, in which some citizens experienced extreme optimism due to the renewed opportunities to go out of their house, even though for a limited range of reasons, while others reckoned that due to the still ongoing pandemic their daily life would not be the same anymore. The latter may have either felt abandoned and exposed by the relevant institutions, who eased stay-at-home orders while providing less strict protective measures to be followed, or endeavored to determine different, feasible, and safe opportunities and habits. Consistently, the second research question for this study will be:

*RQ2*: Do the relevant strategies which compounded reorientation processes during the nationwide COVID-19-related lockdown differ between a time when individuals were adapting to strict stay-at-home orders (T1) and a time when they were getting ready for those orders to be eased in a few weeks even though the pandemic and the related risks were not over yet (T2)?

## 3. Materials and Methods

### 3.1. Participants and Procedures

Data were collected in forms of diary entries during two separate weeks of Italian nationwide lockdown (25–31 March, when participants were adapting to strict stay-at-home orders, and 22–28 April, when they were preparing for those orders to be eased in a few weeks). Participants were asked to submit a daily diary entry, resulting in 14 documents per participant. The stimulus for all the entries was “Could you describe your daily experience and feelings?”. All the involved individuals consented to take part in the study and submitted all the required entries.

Participants included 36 Italian young adults (89% female). Age ranged between 21 and 25 (*M* = 23.02; *SD* = 0.94). All participants attended a Community Psychology course as a part of their master’s degree program at the University of Naples Federico II during the first months of COVID-19 outburst. They all lived in the Campania region. Most of them spent the months of lockdown living with their family (91%), while the others were equally distributed (3% each) between living alone, living with their partner, and living with housemates.

### 3.2. Data Analysis

The Grounded Theory (GT) framework was used. It is a comparative, systemic, approach to collect, analyze, and discuss data [21,22] according to the aim of generating of a situated theory about a phenomenon building on the gathered data [23]. The corpus of data comprised 504 texts, produced by the participants following the above-mentioned stimulus.

**Figure 1:**
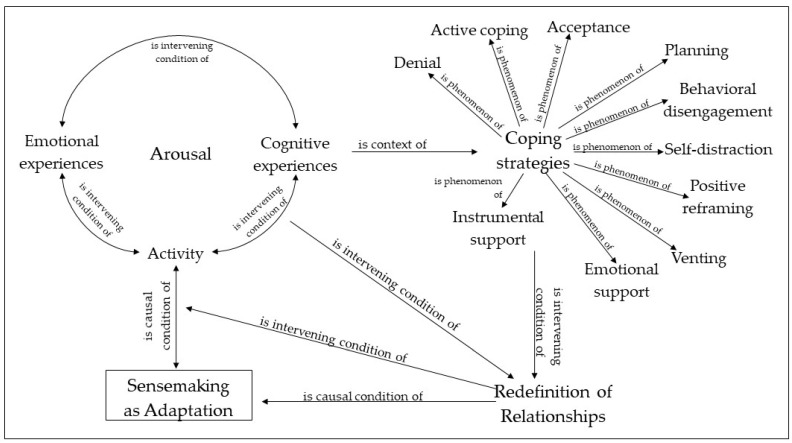
Sensemaking process under lockdown measures.

ATLAS.ti software was used for data analyses, which was run following several different phases as defined by Strauss and Corbin [23]. First, through an open coding, codes were selected in vivo from the corpus as an attempt to represent the main topics and key aspects expressed by the subjects. Then, they were grouped into categories and macro-categories (where needed). Such categories comprise codes sharing common properties and conceptualize and define aspects of the phenomenon. Thus, they express concrete characteristics that recur in the text as well as the relationships among codes and constitute sensitizing elements as they can provide a meaningful narrative of participants’ experience [22]. The second phase is the axial coding, which requires reorganizing data in order to detect the connections and relationships among the identified categories, that is, at a higher level of abstraction. This coding is carried out by referring to a paradigm through which the categories are linked to a macro-category and among them in a set of relationships indicating the causal conditions, the phenomenon, the context, the intervention conditions, and the strategies of action/interaction and consequences. Lastly, the phase of selective coding is aimed at identifying the core category, which is considered central as to the studied phenomenon and in interaction with all the other emergent categories. All the agreed relationships among categories, macro-categories, and the core category are represented in Figure 1. Throughout all the above-mentioned phases, the second and the third authors independently worked and then compared their codes in order to achieve agreement. When this was not possible, the first author intervened for it.

Furthermore, in order to detect differences or similarities in the elements driving reorientation processes when participants were adapting to strict stay-at-home orders (T1) and when they were preparing for those orders to be eased in a few weeks (T2), a mean score was derived for each category by counting how many codes had been categorized in that category in each time, divided by the total number of codes in that category. The scores obtained for each category in T1 and T2 were compared through a repeated measures *t*-test [24].

## 4. Results

Overall, 3147 codes were extracted from the corpus. They were grouped into fifteen main categories (see Table 1): activity, emotional experiences, and cognitive experiences, which were grouped into the macro-category arousal; redefinition of relationships, which was a standing-alone category; active coping, acceptance, planning, denial, behavioral disengagement, self-distraction, positive reframing, venting, emotional support, instrumental support, which were grouped into the macro-category coping strategies, according to Carver’s model [25].

The core category was sensemaking as adaptation, which emerged as the process able to link psychological experience and the need for a redefinition of relationships that stemmed from lockdown experience (see Figure 1). As will be further discussed, this process has been depicted as an iterative and multi-directional one, in which individual psychological experience, that is, arousal and coping strategies, and the social relationships in which individuals are involved contribute to the production of individual and shared meanings about the collective experience of lockdown.

### 4.1. Arousal

The contents of the first macro-category refer to arousal processes, by specifically addressing activity, emotional experience, and cognitive experience—that is, the three main dimensions comprised by this process, that influence each other in a circular relationship [26]. Arousal works as a preliminary sensemaking buffer, not yet crystalizing beliefs and coping strategies, but offering individuals the opportunity to adapt and critically live their own experience. Respondents seemed aware of the relevance of giving meaning to such a peculiar experience, that could have a potential value to their lives both due to its unprecedented characteristics and by offering opportunities for a renewed, and different focus on previous habits, relationships, and activities. They reckoned that the lockdown experience is not part of everyone’s lives, and it was not yet expected to be an event it is important to make the most out of.

*“In these days, I realized that I could live without many things, I could even do without the extraordinary for a while, but today even the word “ordinary” seems to have a new meaning.” M.M., f, 23, t1*

*“What is essential for us? The affections, the family, the friends, the people we love and who love us in turn. […] We never imagine that what we gave for certain and sure could vanish at any moment, yet it happens. Yet it happened.” I.S., f, 24, t1*

Overall, in the face of the COVID-19-related lockdown, respondents reckoned how their ways of experiencing the passing of time had changed. Specifically, they mentioned how time had stopped for them—as if it had been frozen—due to the sudden loss of daily activities and routines. They felt like they were waiting for better times to get on with their lives.

*“In this quarantine it seems that time has stopped, I can hardly distinguish the days that seem to resemble each other more and more unlike before where every single moment, day, hour and minute of our life was marked by work and school commitments, family or moments of simple relaxation.” F.A.T., f, 25, t2*

In the face of this experience, participants engaged in three different ways of physical activation while complying with the sudden stay-at-home orders: they either engaged in indoor, outdoor, or gardening activities when possible (e.g., when gardens or open fields were available as they lived in the countryside), escaped/self-distracted via digital technologies—which were used not only as a tool to keep in touch with others out of the households, but also as means for personal entertainment (e.g., watching movies or reading books)—, and became interested in craft activities. These strategies helped individuals in processing their emotions and cognitions as well as in fulfilling their daytime within their houses.

*“A strange adrenaline pushes me to solve small problems at home.” S.M., f, 23, t2*

*“I had my room clean […] It helps me organize ideas.” E.S., f, 21, t1*

*“Filling in those empty spaces of the day that previously had passed around with friends, in a café, a bookshop, on the streets, etc.” F.P., f, 24, t1*

Indeed, on the one hand, emotional experiences represent a primitive and transient organization of meaning which can be understood as a complex system of evaluation embodied by individuals [27,28,29]. Emotional arousal processes fuel individual behaviors by providing an affective evaluation of what happens in the external environment [30]. As to lockdown experiences, feelings of captivity and of being overwhelmed by one’s own thoughts and emotions emerged among participants, suggesting that the experience they were living represented a burden to them. Further, finding themselves partially or totally distanced from their significant ones could have make them feel lonely and perceive such experiences as even harder to be endured.

*“The bed is where my thoughts and emotions manage to suffocate me.” P.F., f, 22, t1*

These feelings highlight the processual overload due to the need to engage in activities able to fulfill their body and mind, in order to avoid the sense of stasis in which they were immersed, so much so as to remember drowning. Nevertheless, some breaches of hope and positive thoughts emerged too from participants’ words by metaphors referring to nature, which was the only thing not frozen by stay-at-home orders and that was rather benefitting from them. Overall, this suggests that, along with the thoughts about the pandemic and the related social distancing, participants were also attempting to metabolize the distress linked to the forced as well as needed restructuration of their daily habits.

*“I envy them [the birds] a little now, but we will fly together again.” C.G., f, 24, t1*

*“And we are all looking out the window, looking for the rainbow as soon as we see that the rain stops falling, because it reminds us that at the end of everything there is always something beautiful that we can get.” B.S., f, 23, t1*

Further, the feelings of overload made participants feel like time was not enough even now that they were stuck in their houses and the world was somehow frozen, making them often feel exhausted and late. Nevertheless, they acknowledged the paradox intrinsic in such feelings and experiences, which were made even more paradoxical by the perception that days were getting longer in comparison to pre-pandemic times, which was probably due to the lack of activities and frenzy fulfilling daytime during the lockdown.

*“Paradoxically, I have the feeling that time is always too short, even in this circumstance.” N.L., f, 22, t2*

However, while often feeling overwhelmed and overloaded, participants also acknowledged the value such experiences could have for them, due to their being forcedly socially distanced rather than “lost” in the frenzy of their daily lives and of the surrounding world.

*“I wish it would end up returning to normal and I wish it would not end up not getting lost again in the outside world that consumes us.” N.M., f, 22, t2*

On the other hand, cognitive processes allow to selectively collect and process pertinent pieces of information in order to organize and coordinate action schemes in the face of acknowledged changes within the individuals or in their surrounding environment [31]. This cognitive dimension becomes central under emergency circumstances, which are always characterized by changes of different magnitude, as it activates a systematic response of communication, coordination, and control [32,33].

*“I think of the future, of the transformations, of the environmental catastrophes we are destined to.” P.F., f, 22, t1*

This also includes the experience of continuity of personal identity by keeping one’s habits when possible and by relying on memories, which were felt as a key element in order to preserve the unity of one’s identity and to connect the latter to what is happening and its individual and social foreseen consequences.

*“I also like to see the potential for positivity given by the possibility of being able to continue our university path with online lessons that gives me back, at least in part, a piece of my identity as a student, of something that belongs to me and that acts as a bridge between before and now.” B.S, f, 23, t1*

In a downtime where time seemed frozen, the past only constituted memories that seemed far away, and the future was always uncertain. Participants no longer felt able to envision their future selves and lives. However, they detected strategies aimed at keeping track of their plans and meetings for the future in a way that allowed the flexibility needed to sustain those uncertain times, e.g., writing them down but using a pencil.

*“Anchored to the present and to the past, little projected in the future.” R.A., m, 23, t2*

*“I am writing the next plans and appointments using the pencil, for fear of having to further postpone or cancel them.” F.P., f, 24, t1*

Consistently, diary activities were detected as a tool able to help in preserving a sense of continuity and detecting the changes they went through during the lockdown too. Participants noticed the opportunities stemming from this activity and expressed appreciation for the latter as a way to keep track of the small changes they went through day by day and of their emotions.

*“Re-reading myself from the beginning of the first week [referred to the photo-diary] I notice a strong change has been in place.” E.S., f, 22, t2*

*“It has become a reason for me to put pen to paper […] my emotions and thoughts day by day. On the one hand, it allowed me to carve out a moment of my own; on the other hand, to have a trace of it and therefore to re-read myself and perceive the change.” S.M., f, 23, t1*

### 4.2. Coping Strategies

In the face of these sudden cognitive, emotional, and physical activations during a time of relative immobility, and tightly connected to these aspects, several adaptive (or maladaptive) strategies were put in place by the participants to face these unprecedented circumstances and the emergency broadly speaking. That is, participants referred to several coping strategies they adopted to face these unprecedented stay-at-home orders. Building on Carver’s model [25], which provides theoretically driven categories of coping, the identified strategies were here labelled as active, planning, positive reframing, acceptance, emotional support, instrumental support, self-distraction, denial, venting, and behavioral disengagement. Overall, both problem-focused (e.g., active, planning coping strategies) and emotion-focused (e.g., self-distraction and denial coping strategies) strategies [34,35] emerged.

Specifically, participants reported the relief they felt from providing themselves with a better organization about daily activities and tasks, commitments, and obligations. This kind of coping represented an active strategy, that is, focused on doing something about the circumstances one finds oneself in, in order to improve them as far as possible.

*“During these days, it has been nice and useful to be able to stop and process my emotions, due to this continuously changing phase. It has been a moment to cadence and organize my days.” M.R., f, 23, t2*

Furthermore, in order to contrast the current immobility and the inability to continue their normal daily life, participants also extended this new attitude to planning things to do after the end of the lockdown, which can be considered an example of planning coping strategies, that is, thinking about the steps to take in present and future times.

*“I also found myself writing a list of the things I would like to do once the emergency is over.” R.V., f, 23, t2*

Along with planning current and future activities and commitments, participants endeavored to self-distract, even just temporarily, from the “prison” they were living in and from what was happening out of it by engaging in activities which were still feasible despite the stay-at-home orders. Indeed, self-distraction is a coping strategy aimed at self-engaging in activities and thoughts with the aim of take one’s mind off the stressor (in this case, lockdown restrictions and experience).

*“[Writing] gives me the opportunity to feel me less in a cage and shifts my thought away, even though just for a moment.” D.A., f, 25, t1*

*“Many times, I find refuge in words, in readings, and I try to escape a little from everything and everyone in this way.” S.Z., f, 24, t1*

They also attempted to self-distract by imagining scenarios different from the current one, that is, characterized by higher rates of freedom, openness, and chances to take a breath.

*“The game of the week is «Who imagines the most wins» and so I’m trying to put my maximum effort into it. Sometimes it is something I put in place alone, yet sometimes it involves my friends and we imagine together that we are on the rocks in Posillipo [a neighborhood in Naples, Italy]—«Who prepares the sandwiches?»—or about to take a plane—«Where are we going?», «What do you think of this hotel?».” M.M., f, 23, t1*

However, some of the newly set actions and habits also shaped as venting coping strategies—that is, expressing one’s negative feelings via maladaptive behaviors or words—or even behavioral disengagement—that is, giving up the attempt to cope with the stressor and resigning to it.

*“Stress in my life always increased the food quantity.” P.F., f, 22, t1*

Participants also attempted to deny what was happening by refusing to think about it and believe it. They took distances from their new reality also by considering it as something “surreal, movie-like”. This attitude may also have been due to the strong uncertainty they felt about their future and to the evolution of the pandemic, which represented an unprecedented event with uncertain developments to them.

*“Now this is my life, and the moments when I truly think about this new reality of mine are rare.” N.L., f, 22, t2*

*“To date I don’t know how the situation will evolve. Honestly, I also try to think about it as little as possible.” N.M., f, 22, t1*

Conversely, other participants endeavored to adapt to the newness of lockdown daily life and to the changes that would have been brought about by COVID-19 in their lives even after the lockdown, sometimes even detecting and stressing the positive effects of such changes. These have been respectively coded as acceptance—that is, acknowledging that things have changed and endeavoring to learn how to live with the new ones—and positive reframing—that is, attempting to look at things differently in order to focus on their positive side—coping strategies.

*“Adapting to the new modality I don’t like, but better than nothing, I’ll accept the bright sides.” C.G., f, 24, t1*

*“I also feel gratitude, because I feel to thank university and the online approaches which granted us to not loose lessons and not be left behind.” A.L., f, 24, t2*

Participants highlighted the adaptive role of instrumental and emotional support, that is, respectively, getting concrete help and advice, and emotional closeness and understanding from others. On the one hand participants discovered new ways to reciprocally support and share daily activities and habits with their households, while on the other hand they also came up with the discovery of being able to work together and co-operate despite the physical distance, that is, by means of new technologies. Indeed, belonging to a community or other meaningful social groups provides individuals with a sense of meaning and continuity, belonging and safety, affirmation and mattering, even when the connection to such a group is technology-mediated [7,36,37,38,39,40,41].

*“With my great surprise, I discovered that working out together is fun even on videocall.” F.F., f, 22, t1*

*“I have more time to help mom around the house, […] I taught grandma how to make videocalls, I started training with my sister.” E.D., f, 23, t1*

### 4.3. Redefinition of Relationships

In addition to these intrapersonal processes, changes in interpersonal relationships also happened due to stay-at-home orders [12]. Indeed, these orders brought about disruptive changes in the opportunities to relate with others out of the households and to go on with one’s offline social life. Therefore, another category refers to the changes that happened as regards the re-shaping of how people kept in touch with others and valued with whom to do so. Consistently, how much participants missed their daily social habits and routines clearly emerged from their diaries.

*“Today I would like all the ordinary things back, the coffee at the bar, a hug from my best friend, the chats with my friends that always turn into long sleepless nights.” M.M., f, 23, t1*

However, this unexpected break of previous social habits made individuals more aware about the meaning of their significant relationships and the role they played in their lives, up to providing stronger beliefs about how such relationships could be able to overcome forced distances and still provide closeness, warmth, and support.

*“Today, I think about emotional ties, how important they are in this very difficult moment, and how much the distance can be shortened by the good and the love that one feels for the people dear to us.” A.L., f, 24, t1*

In the scenario brought about by stay-at-home orders, digital technologies supported relational habits and social contacts by allowing to keep in touch with significant ones out of the households while complying with the needed measures. This was perceived by participants as a way to “shorten the distances” set by COVID-19-related measures.

*“Technology allows us to shorten distances by making us relive the joy of meeting, confirming that real relationships can be strengthened even more in difficult moments.” M.V.P., f, 23, t1*

Indeed, social media engaged people in sharing their daily life, in a virtualized self-giving way, helping to tolerate the need for postponing in-person meetings, building a deferred in-person encounter perspective over time, along with a feeble sense of certainty. Overall, they were perceived as valid alternatives to cope with negative feelings and process them.

*“I feel relieved when I experience moments of melancholy and I know I can video call my friends.” N.M., f, 22, t1*

*“I listened to the voices of my colleagues, of the professors, because the topics we discussed were not trivial; on the contrary, I admit that they helped me to elaborate some emotional block due to worries or anxiety.” C.G., f, 24, t2*

Nevertheless, while being aware of technologies enabling distant people to keep in touch and maintain their significant relationships, participants were also aware of the sensory limitation of that tool.

*“Having a screen acting as an intermediary for communications makes everything less immediate and spontaneous.” A.D., f, 23, t1*

*“We have many means at our disposal to communicate with friends, relatives, this is good and helps us, yet nothing (luckily!) can really replace a hug when we are down. Today I would like a hug.” S.M., f, 23, t1*

With reference to this, they talked about a “bubble feeling”, an expression that recalls what Hall [42] defined as the proximity space around individuals not accessible at will by everyone. Thus, this expression condenses the need to keep distance from others to avoid contagion and to comply with the mandatory measures issued by local and National Institutions and health authorities, but it also expresses the stemming experiences of isolation, loneliness, and fear generated by these COVID-19-related measures [43,44].

Along with the need to find different ways to keep in touch with significant ones out of the households, participants also experienced another level of relationship, that is the one within the core in which they were locked, which highlighted the need and opportunity to find new ways to live with their households too. Indeed, differently from what happened as to their relationships with their friends and colleagues, in this case they were forced to share house spaces for an amount of hours which were much greater than before COVID-19 outbreak. Nevertheless, this allowed them to detect and experience new activities and hobbies to share in order to enjoy their time together and feel close to each other.

*“I found myself with my fingers wet with coffee and cocoa and I liked it, I enjoyed it. I did not do it all by myself, my father helped me […]. It was a moment of sharing, something we had not done for years, something that united us even more. I had the opportunity and the pleasure of doing something for the first time, staying at home, and I really didn’t think it was possible.” I.S., f, 24, t1*

Among these activities and hobbies, cooking ones clearly stemmed. They were perceived as having twofold benefits. On the one hand, participants perceived these activities as expressions of the cohesion of their family, which proved able to huddle and provide closeness and emotional support to its members. On the other hand, reckoning that these activities were common to most Italian families, as they were broadcasted by National and local television news, made participants perceive cooking activities also as a sort of identity element.

*“Cooking staples, only to discover that many other Italians in this period are also dedicating themselves to cooking pizzas and desserts, to pass the time, heartened me and made me think that in these little things we can see a greater unity in how much nation, how much we would not show in front of the tricolor [Italian flag].” F.P., f, 24, t1*

This was felt as a way to convey the unity of the nation in a time when it was not possible to do it otherwise. Indeed, under collective emergency circumstances, this perception of a common fate can strengthen the sense of belonging to the community, the identification with it, and the tie to it, ending up in higher social cohesion [45,46,47,48].

### 4.4. Comparisons between T1 and T2

As to the repeated measures t-test, the mean scores for T1, that is, during the third week of lockdown, and T2, that is, during the penultimate week before the ease of lockdown measures, and the results of the comparisons are presented in Table 2. Some differences emerged in all the macro-categories, but only with specific reference to some categories.

First, as to the arousal, only emotional experiences showed some differences between T1 and T2, with participants referring more to this area in T1. This may suggest that in T1 participants were more focused on their emotional experience because it was more relevant and fuller of meaning due to the newness of the circumstances they were living in. Conversely, it also suggests a twofold path as to T2: indeed, it is possible either that participants did not perceive the approaching end of stay-at-home orders as something new—thus, their emotional activation was lower—or that in the face of the approaching end of stay-at-home orders some other aspects of participants’ experience emerged as more relevant than their emotional activation, drawing their attention and the focus of their diaries.

*“Today my mind is taciturn, I feel nostalgia and I have every intention of welcoming this emotion. I want to keep it.” M.S., f, 23, t1*

This also reflects in a twofold connotation they attributed to their houses, which were felt either as weapons to fight against the virus and as prisons where citizens got trapped without notice nor explanation nor expiration date according to the times of writing. This may suggest that in the face of a not-yet-known enemy, such as COVID-19 in the first phase of the emergency, being locked in one’s house seemed like the best defense strategy under the hold of emotions such as fear and bewilderment. Nevertheless, over time, houses ended up being framed as overwhelming constraints more than comfortable refuges in citizens’ experience.

*“The houses in which we retire are our weapons.” B.S., f, 22, t1*

*“We found ourselves at home, a bit like prisoners, even if they granted us some yard time.” M.R., f, 23, t2*

Indeed, when the news about the ease of stay-at-home orders was spread (that is, in T2), citizens experienced disorientation, confusion, and worry. On the one hand, they got used to a different organization of their work/study and leisure times and activities and going back to in person activities would have required further changes and planning. On the other hand, they reckoned that their war against the virus was not over yet and that others could still represent potential carriers for the virus to reach their houses and families.

*“4th May is approaching; it is the beginning of a long and worrying coexistence with the virus.” E.D., f, 21, t2*

Consistently with this, it seems possible to hypothesize that in T2 participants were more focused on how to cope with the fatigue due to the months of lockdown and, at the same time, with the bewilderment due to the ease of such measure. Indeed, as to coping strategies, differences emerged with reference to behavioral disengagement and positive reframing strategies. While the first kind of strategy was more frequently referred to in T2, the second one was more frequent in T1. Overall, these differences suggest that along with other coping strategies, during the third week of lockdown (T1) participants relied more on focusing on the positive consequences of lockdown measures and less on resigning themselves to the latter in order to cope with stay-at-home orders and the changes they had brought about in their daily lives and routines. An example of this attitude among Italian citizens can be detected in them singing together from their balconies, exposing billboards stating “everything will be fine” out of their houses, and valuing more the collective dimensions of belonging to a community, sharing a national identity, and providing reciprocal support as far as it was possible in compliance with the protective measures [8,12,49]. Conversely, during the penultimate week before the ease of lockdown measures (T2), that is, after about two months of lockdown, this trend was reversed, with respondents showing higher rates of resignation and lower capacity for positive reframing. A possible explanation for this may lie in participants experiencing higher rates of lockdown- and social-distancing-related fatigue [50] as the day passed by and stay-at-home orders remained in force. Consistently, while during the first weeks they were less tired and more prone to try to find some bright sides of the circumstances they found themselves in, during the last weeks of lockdown they were growing tired and thus were more prone to give up and resign themselves to COVID-19-related changes in their lives and routines.

*“I have to admit I like this peace and the silence that were missing in my previous routines.” G.C., m, 24, t1*

*“I feel almost always alienated in this time, like I could not reach reality anymore, like if I were somewhere else, as if I were living a dream, a film… Could it be because I have never lived an experience like this?” N.M., f, 22, t2*

Last, as to relationship redefinition, participants wrote about it more frequently in T1. This seems is consistent with the need to redefine the opportunities and ways to keep in touch with others both in and out of one’s house in order to maintain one’s significant relationships despite stay-at-home orders, which implied both a forced co-habitation among households and a forced distance among non-households. Indeed, on the one hand, participants experienced a strong need to negotiate spaces, routines, and times within their houses, in order to find a balance in their forced time with their households—be them relatives or not. On the other hand, they also had to find a new balance in how to keep in touch with their significant others not living with them, since all the opportunities for face-to-face contacts unexpectedly disappeared. Conversely, in T2 this issue got less attention, which makes sense—participants were already used to technology-mediated ways of managing their significant relationships and to a new balance of spaces and times inside their house—yet also suggests that the information about the ease of stay-at-home orders had no effect on them with reference to this topic.

*“I miss my days full of moving from one part of the city to another, full of faces, words, I miss the chaotic noise of my city, the voices of friends in sync with the movements of their faces, the human warmth.” S.M., f, 23, t1*

*“It is precisely in our homes that distance is opposed by proximity. These are times when we can experience the joy of having our whole family reunited. We can have lunch together, prepare a dessert together, hear the reassuring footsteps of our mother or father upstairs. I was used to staying at home with my grandfather to study while my parents were at work and my brother at school. Now I am used to seeing them in every room, and it is pleasant, comforting, to the point that if my mother goes out shopping, I miss them.” G.B., f, 22, t1*

## 5. The Core Category: Sensemaking as Adaptation

What emerged from this study represents sensemaking processes about the unprecedented and unexpected experience in which participants found themselves. Sensemaking is described as a process concerning identity and meaning building with reference to the social context one is embedded into, from which people gather cues and with which they exchange perspectives about ongoing phenomena and circumstances; it provides people with more stability and motives their actions [48], helping them adapting to new circumstances—e.g., during the lockdown. Thus, the core category that emerged was defined as “sensemaking as adaptation”, referring to an iterative and multi-directional process linking individual psychological experience, that is, arousal processes and coping strategies, and their redefinition of relationships stemming from lockdown and pandemic experience. Such a process was aimed at producing individual and shared meanings about pandemic-related circumstances, which showed up as unexpected and unprecedented and broke all the already-known knowledge structures and sensemaking processes [51,52]. Indeed, giving meaning to these new and unprecedented circumstances, one finds oneself clearly observing a strong need, consistent with the acknowledgement that it can help in integrating complex experiences into one’s identity, preserving the continuity of the latter [51,52].

*“Such an atypical experience cannot be empty.” A.F., f, 23, t2*

The first phase of sensemaking processes under these circumstances was characterized by emotional answers, followed by the acceptance of the new lack of aspects that characterized participants’ relational and personal life and routines, and the subsequent activation of cognitive processes and physical activities aimed at redefining what was happening and its consequences in terms of daily life. COVID-19 was compared to an invisible enemy that attacked citizens and communities all around the world and against which the latter were fighting a war [53]. Indeed, during the first months after COVID-19 outbreak the ways of transmission of the infection and how to reckon the first symptoms were not yet clear, a circumstance which made the invisibility of this enemy even more worrying.

*“Fear of being altered by an invisible enemy, a collective trauma which will rewrite forever our history.” G.T., f, 22, t1*

*“The news from the front is added, my uncle is a doctor and is working in intensive care.” I.S., f, 24, t1*

Under stay-at-home orders, participants clung to already-known phenomena and circumstances as an attempt to give meaning to the unprecedented experiences they were living by relying on already-known meaning structures and organizations. Indeed, building individual and shared meanings and reorganizing previous ones represent core dimensions when this process happens under disruptive, emergency circumstances [15,51,52], especially during a global disaster that breaks all the already-known certainties and meanings [51,52], producing a broad disorientation [17] due to the loss of daily place and time landmarks and by the subsequent disruption of the meanings traditionally attributed to home, places, relationships, and identities [12]. Specifically, the emotional, cognitive, and physical activations which compounded the first phase of sensemaking processes during the COVID-19-related lockdown gave new value and meaning to individuals’ life projects and relationships and at the same time allowed to acknowledge that appropriate time and space were not dedicated to them before the COVID-19 pandemic due to the hectic pace of everyday life.

This broad redefinition allowed to rediscover and use several coping strategies which relied both on participants’ own characteristics and resources and on their significant social relationships. Indeed, due to the impossibility to go out, citizens could experience an intensive and prolonged cohabitation with the households, which brought about opportunities for a positive reappraisal of family relationships too. These relationships were lived as elusive ones before COVID-19 outbreak, yet due to stay-at-home orders cooperation and active participation for shared aims, as well as reciprocal support and common coping strategies became possible again among households (e.g., cooking together frequently brought about more closeness, support, and opportunities to self-distract for all the involved members). Further, technology-mediated communications and interactions (both via social media and via educational tools and platforms) also allowed individuals to keep in touch with other significant ones and acquaintances, carrying on social as well as educational experiences. They represented the containers that allowed to keep an extra-family sociability under stay-at-home orders.

## 6. Discussion

This study aimed at gaining a better understanding of sensemaking processes during a disruptive and unprecedented event such as the national lockdown that was issued between March and May 2020 as an endeavor to contain COVID-19 pandemic in its very first phase. Specifically, it deepens our understanding of how the familiar elements that represented daily landmarks in pre-COVID-19 routines and daily habits served as reorientation drivers for adaptation and reorientation processes [17] under the new life conditions, that is, under prolonged stay-at-home orders. Such processes acquire further relevance when addressed with reference to young adults, for whom sociability, autonomy, and independence boosts represent critical issues for their evolutionary tasks and identity formation processes [18].

What emerged (see Figure 1) suggests that sensemaking processes went through the acknowledgement of the disruption of daily life and habits as individuals knew them and through the emotional and cognitive processing of such changes, which were associated to the need to engage in the still available activities in order to fulfil the void left by past, now unfeasible, routines. In the face of this acknowledgment, the emotional, cognitive, and activation reactions reflect into several coping strategies played out by relying on both already known and (re-)discovered resources from their houses, referring to instrumental resources as well as to relational ones. This also provided new shapes and meanings to significant relationships, which could now be lived out of the hectic pace of daily life and enjoying the opportunities to share moments and activities, even though in a mediated way, when individuals were not households. That is, individuals had to detect new ways of living together due to the prolonged coexistence of all the households together in the house, yet this also provided them opportunities to experience new ways of supporting each other, being close, and engaging in shared activities. Such a redefinition of the relationships in turn exerted an influence on individuals’ emotional, cognitive, and activation arousal, as well as on the socially rooted processes of sensemaking. In this whole process, emotional reactions, relationship redefinition, and positive reframing coping strategies emerged as more relevant during the first phase of the lockdown, that is, three weeks after it started (T1), while behavioral disengagement coping strategies were more relevant in the last phase of the lockdown, that is, one week before its ease (T2). This may have depended on the need to adapt to new life circumstances when the lockdown started, that is, in T1, as well as on the fatigue which stemmed from the physical and social restrictions brought about by stay-at-home orders after months spent at home [50], that is, in T2.

Overall, this suggests that, in its very first phase, the lockdown was characterized as a liminal experience [16], as individuals endeavored to focus on the positive side of the changes they were forced to face and to adapt their routines and relationships to the new constraints they had to live within. This was probably an attempt to control and reduce their negative emotion. This attitude suggests that this very first phase of COVID-19 pandemic was not experienced as the impact phase of an emergency, as we may have expected, but rather as its remedy or honeymoon phase [19], that is, individuals expressed optimistic viewpoints and relied on primary as well as community relationships and bonding (e.g., using social media). However, it is also necessary to mention that T1 for the present study was in the third week after the enforcement of stay-at-home orders, which means that the impact phase may have been experienced before this time. Conversely, in the last phase of the lockdown this adaptive and proactive attitude left room for resignation to COVID-19 and abandonment of the attempts to cope with it, that is, behavioral disengagement, as it typically happens during the inventory phase [19]. This change in individuals’ attitudes may have depended on two main factors. On the one hand, in the first months of the pandemic, which also represented the first phase of the lockdown, individuals were not aware of how long stay-at-home orders and the pandemic would have taken and were prone to believe the latter would have ended with the ease of the former. Conversely, in the last phase of the lockdown, the need to find ways to live with the virus was clear to everyone as well as the still lacking information about the nature and characteristics of such virus did. On the other hand, almost three months under stay-at-home orders may have produced fatigue and a desire to escape in individuals, who were thus less prone to engage in adaptation and reframing processes and more likely to give up in endeavoring to cope with the changes brought about by COVID-19 pandemic. This may have also been further burdened by the uncertainty about what was going to happen after the ease of lockdown measures, which hindered individuals to further plan how to restart their daily lives and routines. Probably due to this, no element of the present results suggests that citizens were already approaching the reconstruction/recovery phase [19] in the last phase of the lockdown. Indeed, not knowing what was going to happen and how their lives were going to change due to the need to live together with the virus for an indefinite time may have kept citizens back from rebuilding their daily habits and assuming responsibilities for the creation of renewed generative opportunities for themselves and for their community.

Further, the present results suggest that a stronger focus on oneself and on the meanings assumed by one’s own life projects, activities, and relationships, together with the positive reappraisal of primary relationships and social dynamics, represented critical elements to rely on in order to carry on sensemaking processes under prolonged stay-at-home orders. Indeed, relational structures can aid meaning building in sensemaking processes even under social distancing measures by enabling exchanges in the meanings assigned to experiences, providing the opportunity to mobilize inter-individual resources and to face the disaster as a community [15]. Such meanings could help individuals in adapting to and coping with lockdown- and pandemic-related life circumstances at last [15], as they could lead reorientation processes by identifying new boundaries and balances and reconstructing habits and identities in an environment characterized by renewed yet still familiar landmarks [17] during the months of lockdown.

### Limitations and Future Perspectives

The present study offers an exploration of participants’ experience of COVID-19-related lockdown through their emotions, thoughts, and ideas. However, it is necessary to mention that some other variables (e.g., respondents’ mood and rates of anxiety with reference to the overall COVID-19 pandemic and with specific reference to the moments when they wrote their diaries) could have played a role as regards their sensemaking processes and efforts. Further, respondents’ reluctancy in repeating their thought or emotions across close entries and their boredom in writing all the required diary entries should be considered too since they may have resulted in lower commitment in the second part of each week or of the whole study. Last, the results stem from a contextualized and age- and condition- (that is, they were all psychology students) specific experience, which hinders the generalization of these findings to different groups.

Despite these limitations, the present research meets transferability criteria [54] and provides meaningful insights into the psychosocial response to broad disasters such as the COVID-19 pandemic, enriching the established literature with reference to the specific targeted group. Consistently, future studies could broaden these results by deepening our understanding of sensemaking processes with different groups of participants (e.g., of different ages, workers) or even with different approaches building on the present results. Moreover, it could be interesting to further investigate whether some new coping strategies emerged under pandemic circumstances and the consequences each coping strategy brought about in psycho-social terms [25].

Indeed, the pandemic and the subsequent lockdown represented a natural [55] social [56] experiment, as the unprecedented circumstances they brought about allowed to analyze new sensemaking and representations formation processes—in this case, those associated with the outbreak of the virus. Understanding how these processes were shaped during the pandemic and the vulnerabilities and protective factors which associated with them still represents a challenge to be further investigated with future studies. Thus, integrating the present findings with other studies about the later stages of the pandemic would be of great value as to understanding the evolution and consistencies in citizens’ adaptive response and reorientation processes in the face of the COVID-19 pandemic.

## 7. Conclusions

The crisis brought about by the pandemic caused a break in one’s sense of continuity and life planning, determining the need to plan and act under new, uncertain conditions characterized by the lack of landmarks and temporal perspectives [8]. Thus, the need to build new meanings emerged as critical in order to be able to understand and face this crisis [13,57], which was also a crisis of individual and social meanings [3,4]. Building on this, the present study contributes to the understanding of how COVID-19 collective trauma [5] was shaped during the very first months of the pandemic and how individuals attempted to face it while being unprepared and uninformed, by producing new individual and collective meanings and adaptive responses [13]. Under these uncertain circumstances, sensemaking processes were led by emotional reactions first, that helped in building meanings about what was happening.

Since the pandemic and the subsequent lockdown represented a natural [55] social [56] experiment, addressing and understanding what happened under these unprecedented and unexpected circumstances allows to emphasize relational processes, meaning building, and coping strategies to be adopted and how they shaped during a collective trauma [5] which unarmed an entire population worldwide. Specifically, the phases of the pandemic also proved to be different from previous emergencies (e.g., [15,19]) and not to follow a linear trend in the coping strategies to be adopted by citizens, since they have global rather than local effects, show unprecedented characteristics and constraints, and a time pattern does not emerge as to their progression. A deeper understanding of these processes can help in planning adequate interventions and procedures out of those traditionally included in previous experiences of disasters in relation to the phases of emergencies. The adopted strategies reveal the search for meanings, allowing to re-value habits, plans, and relationships, and for a connection between the self and the world outside the house, that can be kept through meaningful relationships, as well as mediated ones. Building contexts allowing this redefinition and a renewed sense of continuity, as well as to maintain meaningful relationships, becomes critical during a syndemic [4]. Building on this, preventive interventions aimed at strengthening the belonging and social fabric of local communities may represent valuable efforts in order to produce communities being able to activate an efficient supportive social network when in need, even when it needs to be activated in a technology-mediated way.

## Figures and Tables

**Table 1:** Summary of codes, categories, and macro-categories.

Macro-Category	Category	Codes (*n*)
Arousal	Activity	98
	Emotional experiences	1420
	Cognitive experiences	490
-	Redefinition of relationships	291
Coping strategies	Active coping	36
	Acceptance	100
	Planning	51
	Denial	27
	Behavioral disengagement	12
	Self-distraction	283
	Positive reframing	283
	Venting	33
	Emotional support	29
	Instrumental support	25

**Table 2:** Mean scores for each category in T1 and T2 and comparisons between the two times of data collection.

Categories	T1		T2		Repeated Measures *t*-Test	
	*M*	*SD*	*M*	*SD*	*t* (*df*)	95% CI
Arousal						
Activity	2.44	1.73	2.30	1.92	0.36 (35)	[−0.64, 0.92]
Emotional experiences	36.86	15.90	30.92	18.62	2.82 ** (35)	[1.68, 10.22]
Cognitive experiences	13.42	6.28	11.55	6.97	1.47 (35)	[−0.70, 4.42]
Redefinition of Relationships	9.86	4.82	7.14	3.88	3.20 ** (35)	[0.99, 4.45]
Coping strategies						
Active Coping	0.86	0.83	0.94	1.24	−0.34 (35)	[−0.58, 0.41]
Acceptance	2.50	1.86	2.75	2.48	−0.58 (35)	[−1.13, 0.63]
Planning	1.25	1.27	1.39	1.93	−0.46 (35)	[−0.75, 0.48]
Denial	0.55	0.84	0.55	0.91	0 (35)	[−0.38, 0.38]
Behavioral Disengagement	0.36	0.68	0.75	0.84	−2.68 * (35)	[−0.68, −0.09]
Self-distraction	7.92	3.25	6.61	3.44	1.71 (35)	[−0.24, 2.86]
Positive Reframing	7.47	3.06	5.83	3.63	2.10 * (35)	[0.05, 3.23]
Venting	0.53	0.97	0.55	0.65	−0.17 (35)	[−0.36, 0.30]
Emotional Support	1.22	1.22	1.17	1.25	0.20 (35)	[−0.51, 0.62]
Instrumental Support	1.25	1.10	1	1.17	0.94 (35)	[−0.29, 0.79]

*Note*. *M* = mean; *SD* = standard deviation; *df* = degrees of freedom; CI = confidence interval. ** *p* < 0.01 (2-tailed); * *p* < 0.05 (2-tailed).

## Data Availability

The data presented in this study are available on request from the corresponding author. The data are not publicly available due to privacy reasons.
